# Study of the Treatment Effects of Compound Tufuling Granules in Hyperuricemic Rats Using Serum Metabolomics

**DOI:** 10.1155/2018/3458185

**Published:** 2018-10-16

**Authors:** Peng Wu, Jing Li, Xianxian Zhang, Fuling Zeng, Yingwan Liu, Weifeng Sun

**Affiliations:** ^1^Department of Traditional Chinese Medicine, General Hospital of Guangzhou Military Command of PLA, Guangzhou 510010, China; ^2^Guangzhou University of Chinese Medicine, Guangzhou 510006, China

## Abstract

The study aimed to investigate the mechanism of the effect of Compound Tufuling Granules (CTG) to lower the serum uric acid level in a rat model of hyperuricemia. The rat model was established by administering hypoxanthine through oral gavage and potassium oxonate through intraperitoneal injection. Rats were divided into the normal group, model group, CTG group, and allopurinol group. Serum uric acid, creatinine, urea nitrogen, and inflammatory cytokine levels were determined in each group. In the model group, ultrahigh performance liquid chromatography-mass spectrometry was used to analyze the metabolic profiles and delineate the action mechanism of CTG; in addition, the orthogonal projection method was used to perform latent structure-discrimination analysis to screen the related metabolites. The results indicated significant differences in the metabolic profiles between the model and normal groups. A total of seven related metabolites were identified through screening in the model group, mainly related to the pathways of bile secretion, pyrimidine, purine, and phenylalanine metabolism, pantothenate and CoA biosynthesis, and pentose and glucuronate interconversions; these related pathways were reversed in the CTG group. In the metabolic networks, uracil and acetyl-coenzyme A were the nodal molecules. In addition, the test results of the evaluation of serum biochemical and inflammatory factors confirmed that CTG had significant effect in reducing the levels of serum uric acid and protecting renal function. These results confirmed that CTG primarily regulated the recruitment of nodal molecules to achieve anti-inflammatory effects, reduced uric acid level, and renal protection.

## 1. Introduction

Hyperuricemia is characterized by persistent increase in the uric acid level in the circulating blood. Oversaturated uric acid in the body may be due to increased uric acid synthesis or decreased excretion, which is the most important factor in the onset of gout [1, 2]. In addition, hyperuricemia is an independent risk factor for other diseases, such as hypertension, diabetes, obesity, hyperlipidemia, and heart disease [3–5]. At present, the incidence of hyperuricemia is increasing worldwide with increased occurrence in younger individuals, leading to a huge economic and social burden [6]. Since humans lack the corresponding enzymes, uricase, and allantoin, in vivo metabolism of purines involves xanthine oxidase-mediated decomposition to xanthines and finally conversion to uric acid [7, 8]. Therefore, lowering the uric acid level is a key to treating patients with hyperuricemia.

At present, the uric acid lowering drugs are mainly classified into two major categories: inhibitors of uric acid production and promoters of uric acid excretion. The drugs that inhibit the production of uric acid mainly include allopurinol and febuxostat, and the main drugs that promote its excretion include benzbromarone and probenecid. Due to their serious side effects, such as serious allergic reactions and rashes caused by allopurinol, the drugs that promote excretion have higher requirement for adequate renal function, with exacerbation under combined usage, which limits the use of these drugs, especially in patients with asymptomatic hyperuricemia [9, 10].Therefore, studies to determine safer and more effective treatments to reduce uric acid levels are needed.

As an effective alternative treatment method, traditional Chinese medicine (TCM) has advantages in the treatment of patients with gout and hyperuricemia. Compound Tufuling Granules (CTG) is one of the representative treatments using TCM [11, 12]. It has been simplified by the experience party Xie-Zhuo-Chu-Bi-Fang and obtained the national invention patent (Patent no.: ZL 200710032363.3). It was formulated into nonstandard military preparation of CTG (lot no: Guang L2009001), consisting of* Rhizoma Smilacis Glabrae*, Rhizoma Dioscoreae Hypoglaucae, Pseudobulbus Cremastrae Seu Pleiones, Radix Achyranthis Bidentatae, and Semen Vaccariae, which has the functions of clearing heat and dampness and activating blood circulation to relieve pain. Previous studies have shown that it lowers the level of uric acid, preventing the recurrence of gout and suppressing the expression of inflammatory factors [13, 14]. However, the pharmacological mechanism of CTG-induced reduction in the uric acid level is unknown to date.

Metabolomics is the study of changes in metabolites produced by biological systems (the cell, tissue, or organism) after they have been stimulated. Small molecules are its target research objective, including their production and metabolism as end result of a series of events; therefore, metabolomics defines the state of biological systems more accurately [8, 12, 15, 16]. Currently, metabolomics is widely used in the field of TCM [7, 11, 17–19]. Commonly used metabolomics techniques include nuclear magnetic resonance (NMR), gas chromatography with mass spectrometry (GC-MS), liquid chromatography with mass spectrometry (LC-MS), ultrahigh performance liquid chromatography-mass spectrometry (UPLC-MS), and other such techniques, with unique advantages and disadvantages. Urine metabolome is more dependent on exogenous factors, such as fluid intake, as compared to serum metabolome, which is less affected by interfering factors and is more stable than the urine metabolome. In general, UPLC-MS is focused on identifying large molecular-weight polar metabolites; moreover, identification of metabolites through UPLC-MS is more effective than that through GC-MS, which does not allow gasification due to the presence of high molecular-weight metabolites; hence, for the purpose of our study, we selected the UPLC-MS detection method [7, 20]. In this study, UPLC-MS technology was used to investigate the serum metabonomics aspect of lowered uric acid levels in rats undergoing treatment with CTG.

In this study, oral hypoxanthine and intraperitoneal injection of potassium oxonate were used to establish the hyperuricemia rat model; the rats were divided into the normal group (N), model group (M), CTG group (Fa), and allopurinol group (B) (positive control group). In each group, the serum creatinine (Scr), serum uric acid (SUA), and blood urea nitrogen (BUN) levels were determined using biochemical tests; the levels of inflammatory cytokines TNF-*α* and IL-1*β* were determined using ELISA method, and for the purpose of serum metabolome study, HPLC-MS was used. The first aim was to elucidate the abnormal metabolic mechanism of hyperuricemia, and the second aim was to identify the relevant specific metabolic markers of uric acid metabolic pathway and other possible metabolic pathways in rats undergoing treatment with CTG, which will enable early diagnosis of patients with hyperuricemia.

## 2. Materials and Methods

### 2.1. Reagents and Instruments

For the purpose of study, 97% oxonic acid potassium salt and 99% hypoxanthine were purchased from Sigma (St. Louis, MO, USA); soluble starch from Shanghai Macklin Biochemical Science and Technology Co., Ltd. (Shanghai, China); ELISA kits for TNF-*α* and IL-1*β* from Beijing Cheng Lin Biological Technology Co., Ltd (Beijing, China); and methanol, chloroform, formic acid, L-2-chlorobenzene alanine, and acetonitrile from Shanghai Heng Chuang Biological Technology Co., Ltd. In addition, we used the following equipment: ultrasonic cell pulverizer and ultrasonic cleaning machine (Scientz-IID and SB-5200DT, respectively; Ningbo Xin Zhi Biologic Multi Tube Technology Co., Ltd. Ningbo, Zhejiang, China); vortex (TYXH-I; Shanghai Khan Novo Instrument Co. Ltd. Shanghai, China); high speed refrigerated centrifuge (TGL-16MS; Shanghai Luxiang Centrifuge Instrument Co., Ltd. Shanghai, China); and UPLC (ACQUITY UPLC I-Class), High resolution mass spectrometry (VION IMS Q-Tof), and chromatographic column (ACQUITY UPLC BEH C18) (100 mm × 2.1 mm, 1.7 um) from Voight World Science and Technology Co., Ltd. (Waters) (Shanghai, China).

### 2.2. CTG Preparation

CTG comprises the granules of the Xiezhuo Chubi formulation, which consists of* Rhizoma Smilacis Glabrae*, Rhizoma Dioscoreae Hypoglaucae, Pseudobulbus Cremastrae Seu Pleiones, Radix Achyranthis Bidentatae, and Semen Vaccariae (Table 1). As per requirement of nonstandard military preparation, CTG was made by the Department of Pharmacy of the Guangzhou Military Command General Hospital (batch number: Guang L2009001), in packets of 10-g weight each (batch No. F01009) [14]. The formulation was extracted and purified by means of water extraction and alcohol sedimentation process [21]. The excipient of the granules included soluble starch (Xiangtan County Starch Products Co., Ltd., batch number: 20110116). The quality of the granules was measured using thin layer chromatography (TLC) and HPLC [22]. Allopurinol tablets provided by the Western Pharmacy Department of the Guangzhou Military Command General Hospital were purchased from Chongqing Qingyang Pharmaceutical Co., Ltd. (Chongqing, China).

### 2.3. Animal Models and Experimental Study Design

The experimental protocols were approved by the ethics committee of the General Hospital of Guangzhou Military Command; all experimental procedures were conducted in accordance with the National Institute of Health guidelines for the care and use of laboratory animals.

Thirty-two SPF-grade SD rats with body weight of 200±20 g provided by the Experimental Animal Center of Southern Medical University were divided into eight cages, of four rats per cage. All included animals were housed and fed in the SPF Animal Experimental Center of the General Hospital in Guangzhou Military Command; the room temperature was maintained at 23±2°C and the humidity was maintained at 55%±5; the rats were maintained on a 12-h light cycle. After adaptive feeding for 7 days, the rats were randomly divided into the N, M, Fa, and B groups at eight rats per group. Except for the N group, oral hypoxanthine 500 mg/kg and intraperitoneal morning injection of potassium oxonate 100 mg/kg were administered to each group for a total of 10 days. The CTG and allopurinol solution was freshly prepared for administration through gavage. The allopurinol tablets and CTG were crushed to powder and subsequently dissolved in distilled water to achieve concentration of 500 mg/ml and 1.25 mg/ml, respectively. The injection volume was 8 ml/kg. The Fa and B groups were administered orally with specific volume of the indicated drugs at 4 g/kg and 5 mg/kg, respectively; the N and M groups were administered orally with the same volume of distilled water. On day 4 of the experiment, the drug-groups were administered CTG or allopurinol solution through gavage at 30 min after injection of the modeling agent. Dosage of all modeling agents, drugs, and distilled water were converted according to the body's surface area [23]. Each administration group received gavage once a day for a total of 7 days; the entire experiment lasted for 10 days.

### 2.4. Metabolomic Sample Collection

At 2 hours after last administration of the drug, blood was collected from the orbital vein of the rat under anesthesia; subsequently, the blood sample was centrifuged at 3000 rpm at 4°C for 5 minutes. The resulting supernatant was centrifuged at 12,000 rpm for 5 minutes to remove insoluble proteins and then stored at -80°C until use in the metabolomic assay.

### 2.5. Serum Biochemical and TNF-*α* and IL-1*β* Assay

Anesthesia was performed through abdominal injection of choral hydrate (350 mg/kg). The blood sample (3 ml) was collected from the abdominal aorta, placed in a red tube (non-anticoagulation tube), allowed to stand at room temperature for 30 minutes, and centrifuged at 3000 rpm for 20 minutes; the obtained serum was sent to the General Hospital of Guangzhou Military Region for laboratory tests including SUA, Scr, and BUN detection. The remaining supernatant was collected for use in ELISA for TNF-*α* and IL-1*β* performed using the manufacturers' instructions.

### 2.6. Sample Preparation for Metabolomics Study

The internal standard (10 *μ*l) (L-2-chlorophenylalanine, 0.3 mg/ml, methanol configuration) was added to serum (100 *μ*l) and the solution was mixed by vortexing for 10 s; subsequently, methanol-acetonitrile (300 *μ*l) (2:1, v/v) was added followed by vortexing for 1 min. Extraction was performed in an ice water bath, and the sample was maintained at -20°C for 30 min; centrifugation was performed at 13,000 rpm for 15 min using a 0.22 *μ*m organic phase pinhole filter to obtain filtrate (supernatant, 200 *μ*l) which was transferred to the liquid chromatography injection vial. The quality control sample (QC) was prepared by mixing equal volume of the extracts of all samples, to obtain the same volume as that of the sample.

### 2.7. UPLC-MS Process

UPLC conditions are as follows: column, ACQUITY BEHC18 column (100 mm × 2.1 mm i.d., 1.7 *µ*m; Waters, Milford, USA); solvent, the column was maintained at 45°C and separation was achieved using the following gradient: one to 30% B over 0–1 min, 30–60% B over 1–2.5 min, 60–90% B over 2.5–6.5 min, and 90–100% B over 6.5–8.5 min; the composition was held at 100 % B for 2.2 min, followed by 10.7–10.8 min, 100% to 1% B, and 10.8–13 min holding at 1% B at flow rate of 0.40 ml/min, where B is acetonitrile/methanol 2/3 (v/v)(0.1% (v/v) formic acid) and A is aqueous formic acid (0.1% (v/v) formic acid). Injection volume was 1 *μ*l and the column temperature was set at 45°C.

The mass spectrometric data was collected using Waters VION IMS Q-TOF mass spectrometer equipped with an electrospray ionization (ESI) source operating in either positive or negative ion mode. The capillary voltages, DP, and CE were 2.5 kV, 40 V, and 6 eV, respectively. Source temperature and desolvation temperature were set at 115°C and 450°C, respectively, with desolvation gas flow at 900 l/h. Centroid data was collected from 50 to 1,000 m/z with scan time of 0.2 s and interscan delay of 0.02 s over a 13-min analysis time-period. The QCs were injected at regular intervals (every 10 samples) throughout the analytical run to provide the data set used for repeatability assessment.

### 2.8. Data Processing and Statistical Analysis

The acquired UPLC-MS raw data were analyzed using the Progenesis QI software (Waters Corporation, Milford, USA) with the following parameters: precursor tolerance of 5 ppm, fragment tolerance of 10 ppm, and retention time (RT) tolerance of 0.02 min. Internal standard detection parameters were deselected for peak RT alignment, isotopic peaks were excluded from the analysis, noise elimination level was set at 10.00, and minimum intensity was set to 15% of base peak intensity. The excel file with three-dimension data sets was obtained, including m/z, peak RT, and peak intensities; RT–m/z pairs were used as the identifier for each ion. The resulting matrix was further reduced by removing any peaks with missing value (ion intensity=0) in more than 60 % samples. The positive and negative data were combined to obtain a combined data set, which was imported into the SIMCA software (version 14.0, Umetrics, Umeå, Sweden). Principal components analysis (PCA) and orthogonal partial least-squares-discriminant analysis (OPLS-DA) were conducted to visualize the metabolic alterations among the experimental groups. Variable importance in the projection (VIP) ranks the overall contribution of each variable to the OPLS-DA model, and those variables with VIP > 1 are considered as relevant for group discrimination. Other data were analyzed using one-way ANOVA followed by the least significant difference (LSD) test. All statistical analyses were performed using SPSS version 20.0 (IBM, Armonk, NY, USA). The results were assumed to be statistically significant at P<0.05.

### 2.9. Related Metabolite Identification

The related metabolites were identified as follows: First, potential metabolites were separated in the loading scatter plot of OPLS-DA (the N and M group). Second, the related metabolites were further screened by limiting VIP (VIP > 1) and performing Student's t-test (p<0.05). The information of these metabolites was obtained through searches conducted in the Kyoto encyclopedia of genes and genomes (http://www.kegg.jp [24]) and HMDB (www.hmdb.ca). Finally, commercial standards were adopted to support the metabolites' identification.

## 3. Results

### 3.1. Serum Biochemical Analysis and ELISA

**Figure 1:**
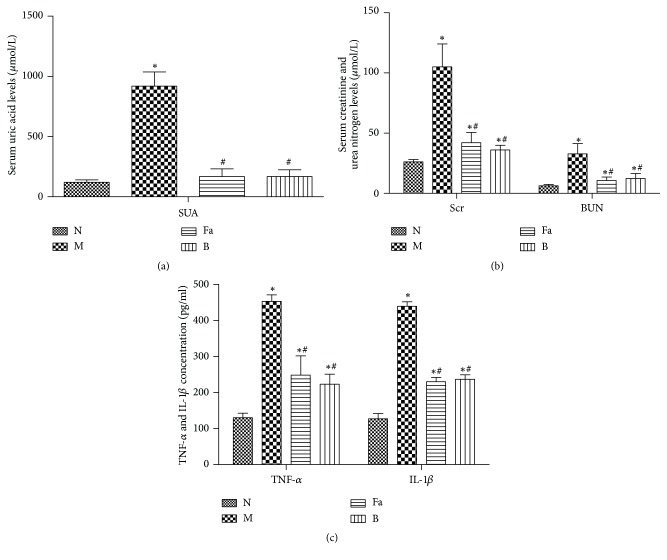
Concentration of SUA (a), Scr, BUN (b), and TNF-*α* and IL-1*β* (c). Compared with the N group, ^∗^p<0.05; compared with the M group, ^#^p<0.05.

As shown in Figure 1, significant increase in the SUA level is an important biochemical basis for the development and detection of hyperuricemia, and impaired kidney function is effectively reflected by the level of Scr and BUN. The involvement of inflammatory factor(s) is an important basis for the onset of gout and hyperuricemia. Compared with the N group, the levels of SUA, Scr, BUN, TNF-*α*, and IL-1*β* in the M group were significantly elevated* (p<0.05). *Compared with the M group, the levels of SUA, Scr, BUN, TNF-*α*, and IL-1*β* in the Fa and B groups were lower than those in the M group* (p<0.05)*, without significant difference between the Fa and B groups* (p>0.05).* These results indicated that the Fa group had reduced level of uric acid, improved renal function, and anti-inflammatory effects.

### 3.2. Serum Metabolic Profiles

**Figure 2:**
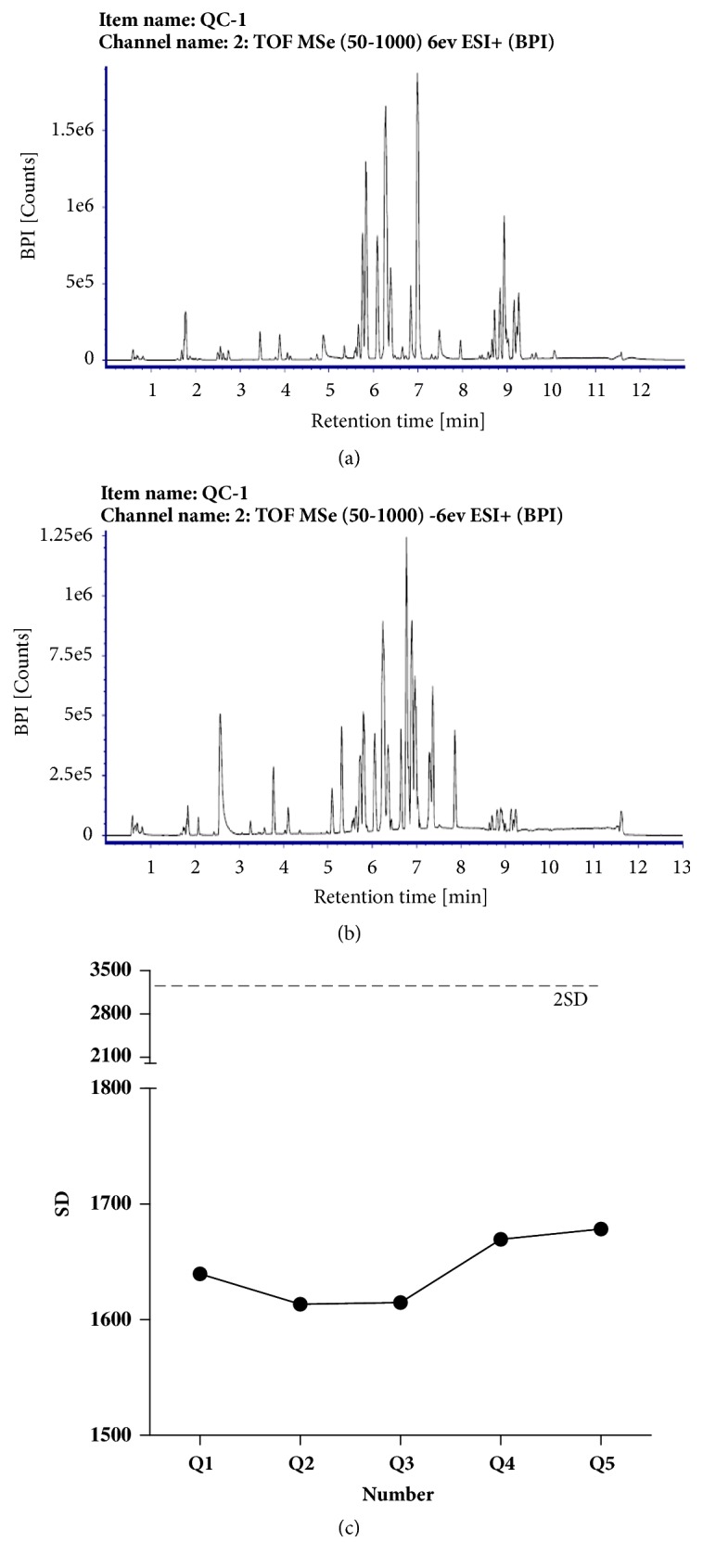
Quality control sample (QC) base peak ion chromatogram and PCA analysis results. (a) Base-band ion flow diagram at positive ion mode of the control sample BPI (+). (b) Base-level ion flow diagram at negative ion mode of the control sample BPI (-). (c) QC plot of five repeat UPLC-MS analyses. X-axis, number, y-axis, standard deviation (SD).

In all samples, the UPLC-MS method was used to detect the base peak ion current (BPI); the instrument analyses were characterized by strong signal, large peak capacity, and good reproducibility of retention time (Figures 2(a) and 2(b)). In the analysis sequence, one QC sample was inserted into every 10 analysis samples. The analyses showed that the method had good repeatability and met the analysis requirements of the metabolic group (Figure 2(c)).

### 3.3. Potential Biomarkers Related to Hyperuricemia

**Figure 3:**
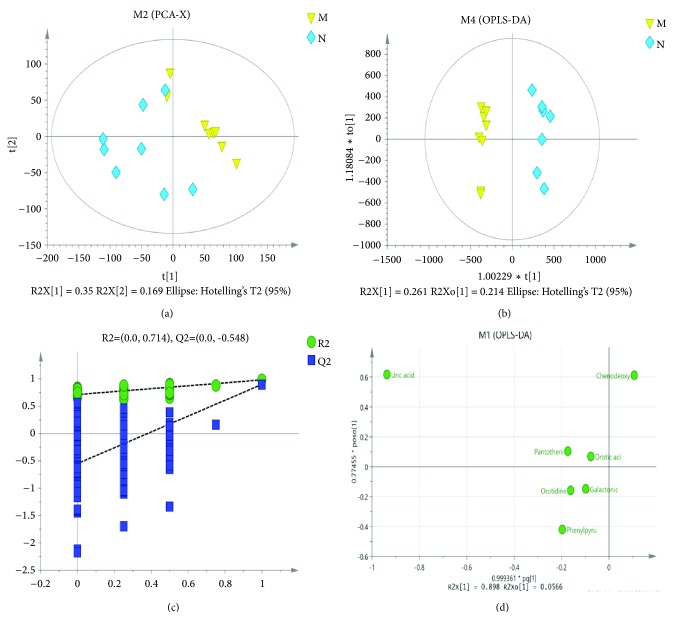
Multivariate data analysis. (a) Principal component analysis (PCA) score map derived from UPLC-MS in the normal rats (*◇*) and hyperuricemia model rats (▽). (b) Orthogonal projections to latent structures-discriminant analysis (OPLS-DA) score. (c) Validation plot obtained from 200 permutation tests. (d) The loading scatter plot of OPLS-DA.

First, PCA was used to analyze rats in the N and M groups. In the PCA model, there were significant differences between the N and M groups (R2X=0.704, Q2=0.49), indicating that the model was reliable (Figure 3(a)). To obtain more reliable related metabolites, we used OPLS-DA to filter the model-independent signals and obtain the final OPLS-DA model. As a result, two categories, R2Y (0.982) and Q2 (0.9), were obtained, indicating that the two groups of samples had significant differences in the OPLS-DA score map (Figure 3(b)). We performed 200 response sequencing tests on the OPLS-DA model (Figure 3(c)) and obtained intercepts of R2=0.879, Q2=−0.331, indicating that the OPLS-DA model was without overfit and reliable. The relevant metabolites were screened by defining VIP>1 for the first principal component of the OPLS-DA model and Student's t-test (p < 0.05). The loading scatter plot of OPLS-DA (Figure 3(d)) and statistical analysis (Table 2) indicated increased levels of galactonic acid, pantothenic acid, orotidine, orotic acid, uric acid, phenylpyruvic acid, and decreased levels of chenodeoxycholic acid.

### 3.4. Effects of Allopurinol and CTG on Metabolite Profiling

**Figure 4:**
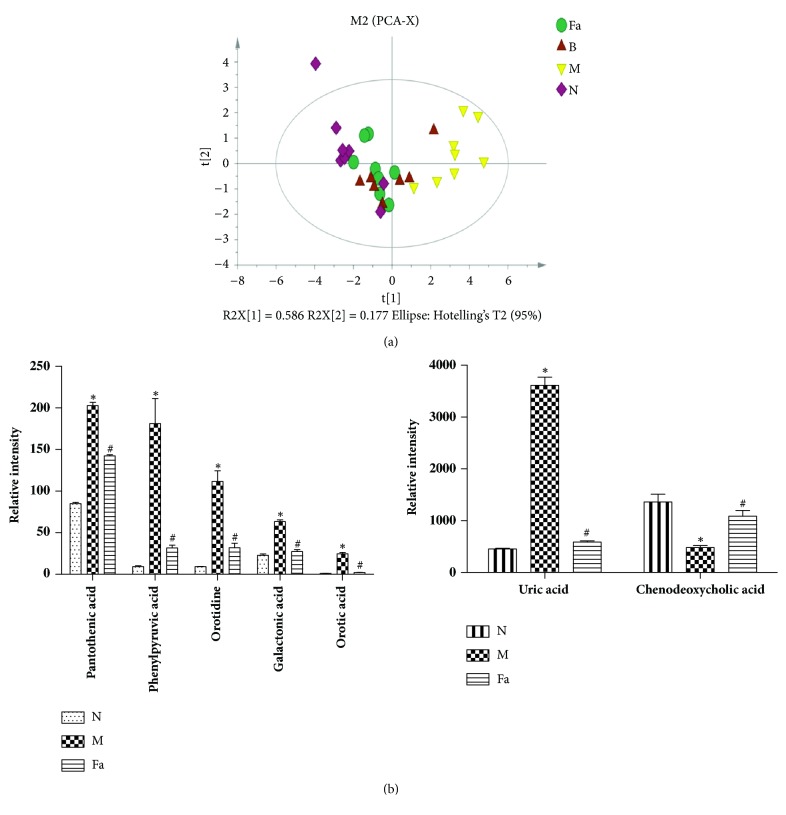
Multivariate data analysis. (a) PCA scores' plot derived from the serum levels of seven metabolites in the normal group (*◇*), hyperuricemia model group (▽), Fa group (○), B group (Δ). (b) Bar plots show UPLC-MS relative intensities for seven metabolites in the normal, model, and Fa groups. Data are expressed as the mean ± SD using one-way ANOVA. Compared with the normal group, ^∗^p<0.05; compared with the model group,^ #^p<0.05.

For purpose of identifying potential drug-treatment target, we established a new PCA model including seven different metabolites. Compared with the model, the B and Fa groups were more similar to the N group (Figure 4(a)), indicating that allopurinol and CTG had capability to reverse the pathological process of hyperuricemia. To further explain the different degree of improvement of the CTG-related seven metabolites, we used one-way analysis of variance* (p<0.05)*. Compared with the model, CTG reduced the expression level of galactonic acid, pantothenic acid, orotidine, orotic acid, uric acid, and phenylpyruvic acid to varying degree and increased that of chenodeoxycholic acid (Figure 4(b))* (p <0.05)*.

### 3.5. Interpretation of Metabolic Networks

**Figure 5:**
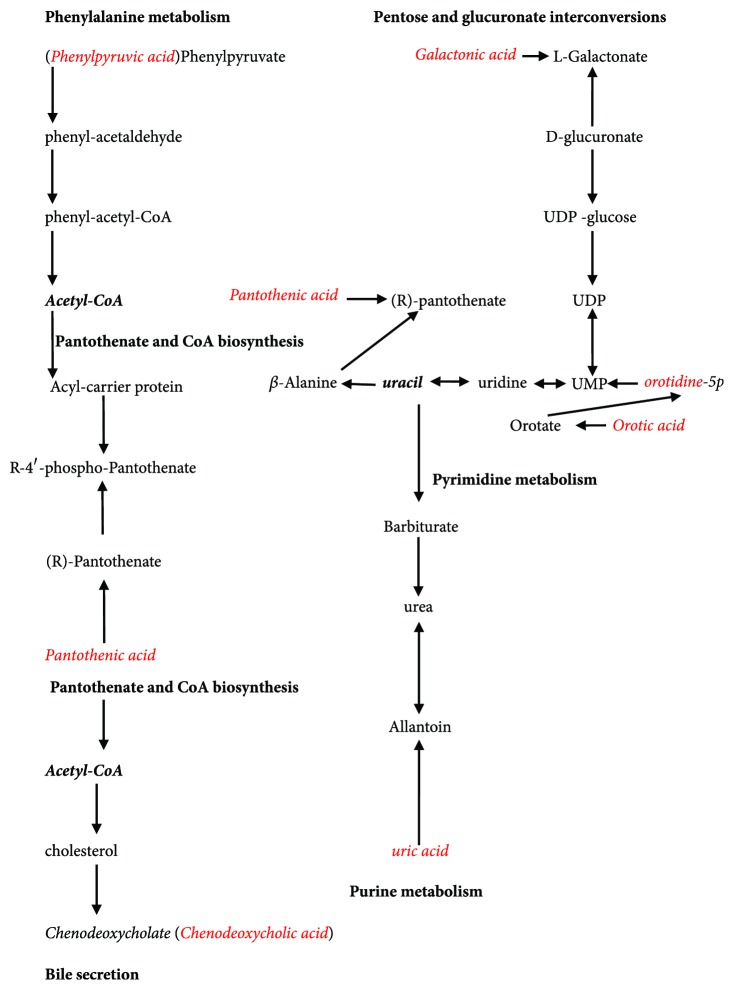
Diagram of the metabolic pathway networks involved in the CTG intervention. The nodal molecules are represented in bold italic font. The seven related metabolites are represented in red font. The pathway names are in bold font.

In this experiment, CTG showed significant effects of anti-inflammation, decreased level of uric acid, and improved renal function at varying degrees. Compared with the N group, the M group included seven related metabolites through the UPLC-MS method. These substances are mainly related to bile secretion, pyrimidine, purine, and phenylalanine metabolism, pantothenate and CoA biosynthesis, and pentose and glucuronate interconversions pathways, which are directly or indirectly related pathways. Uracil, as a nodal molecule, is associated with pyrimidine and purine metabolism, pantothenate and CoA biosynthesis, and pentose and glucuronate interconversions; in addition, acetyl-coenzyme A (acetyl-CoA) is another nodal molecule in the pathways of bile secretion, pantothenate and CoA biosynthesis, and phenylalanine metabolism. In metabolic networks (Figure 5), the core pathways included pyrimidine metabolism and pantothenate and CoA biosynthesis. In this study, the Fa group showed reversed expression levels of the seven related metabolites, indicating that CTG can potentiate the role of other pathways by regulating the nodal molecules in the networks.

## 4. Discussion

Hyperuricemia is closely related to the occurrence of gout, as well as several metabolic diseases such as obesity, diabetes, and hyperlipidemia. Allopurinol inhibits both the synthesis and metabolism of uric acid. By inhibiting xanthine oxidase, it prevents the conversion of hypoxanthine and xanthine to uric acid, thereby reducing the concentration of uric acid in the blood. Previous studies using the rat model of hyperuricemia have shown that CTG inhibited xanthine hydrogenase activity (XO) and xanthine oxidase (XDH) mRNA expression in the liver and anti-inflammation, promoted uric acid excretion by regulating the expression of miR-34a and miR-146a, and inhibited URAT1 and GLUT9 mRNA transcription and protein expression [13, 14, 25, 26]. However, the metabolic mechanism involved remains unclear. In the present study, we conducted serum metabonomics' study in rats with hyperuricemia by means of UPLC-MS.

Increased synthesis or decreased excretion of uric acid in the body can lead to the occurrence of hyperuricemia. Therefore, currently, animal models of hyperuricemia are established using mainly in vitro xanthine/hypoxanthine infusion and in vivo inhibition of the uricase activity [17, 19]. In this study, we successfully prepared the hyperuricemia model by oral gavage of hypoxanthine and intraperitoneal injection of potassium oxonate. A large number of studies have confirmed that long-term increase in the SUA level triggered the acute onset of gout, as well as the deposition of urate crystals in the internal organs, causing renal insufficiency [2, 4]. Secondly, the acute onset of gout was additionally associated with many inflammatory factors; of these, IL-1*β*, a key cytokine of gout, can act on many cell types to initiate inflammatory responses, whereas TNF-*α*, a type of macrophage and pro-inflammatory cytokines produced by monocytes, participates in the inflammatory response of gout. Moreover, studies have confirmed that elevated levels of IL-1*β* and TNF-*α* can be detected in the blood of rats with gout and those of the hyperuricemia model [27, 28]. CTG consists of five herbs. Among them,* Rhizoma Smilacis Glabrae* has the functions of clearing heat and detoxication and promotion of blood flow of the joints and kidney. The main function of Radix Achyranthis Bidentatae is to replenish qi, activate blood circulation, and promote water and uric acid excretion. Rhizoma Dioscoreae Hypoglaucae has the function of dispelling wind and removing dampness and diuresis. Semen Vaccariae has the function of promoting blood circulation and removing blood stasis, promoting metabolism, and improving microcirculation. Colchicine is the active ingredient of Pseudobulbus Cremastrae Seu Pleiones, which significantly reduced the inflammatory reaction [13, 14, 25, 26]. In this study, as compared to the normal range for this species, the levels of SUA, Scr, BUN, IL-1*β*, and TNF-*α* were significantly increased in the rat model, while those in the Fa group were significantly lower, indicating that CTG can reduce the level of uric acid through anti-inflammatory action and improving the renal function.

In clinical studies, the diagnosis of hyperuricemia is based on the SUA level alone, which limits early detection; therefore, identifying significant related metabolites is crucial. In our study, results of screening indicated seven metabolites, mainly related to bile secretion, pyrimidine, purine, and phenylalanine metabolism, pantothenate and CoA biosynthesis, and pentose and glucuronate interconversions.

In metabolic networks, galactonic acid is the product of pentose and glucuronate interconversion through the oxidation of hydroxy groups in galactose to form shuttle groups. Galactose is a type of monosaccharide that can be catalyzed by *β*-glycosylase in the intestine. Galactose for anabolism can be produced from uridine diphosphate glucose (UDPG) in the absence of food substrate. It can be found in dairy products, and plant mucin and bacterial polysaccharides, which is the component of lactose in mammalian milk [29]. More importantly, galactonic acid can be associated with D-glucuronate through L-galactonate and subsequently with pyrimidine metabolism through UDPG. Studies have shown that glucose metabolism is involved in the development of gout and hyperuricemia and hyperuricemia exacerbates the disorder of glucose metabolism [30, 31]. In our study, the level of galactonic acid was upregulated in the M group versus the N group; in addition, the related pathway of pentose and glucuronate interconversions and pyrimidine metabolism were dysregulated. However, as compared with the M group, these effects were reversed in the Fa group, with no significant difference compared with the N group, indicating that CTG can inhibit abnormal glucose transformation in vivo to lower the level of uric acid.

Uric acid is the final metabolic product of purine, one of the most important components of DNA and RNA. Purine is decomposed into uric acid through a series of catalytic reactions; in some animals, under the effect of uricase, it is further decomposed to allantoin and urea through association with the pyrimidine metabolism via uracil [19]. Pyrimidine metabolism comprising orotic acid and orotidine was first core of the hyperuricemia metabolic networks. Orotidine can be decomposed into uridine monophosphate (UMP) and uridine diphosphate (UDP). UMP is further decomposed into uridine and uracil which is a unique base of RNA. In transcription of DNA, thymine (T) in DNA is substituted and paired with adenine to methylate uracil which is an important component of nucleic acids. The results of previous studies confirmed that uridine, uracil, and pyrimidine metabolism had a pathogenic role in the development of hyperuricemia and exacerbation of acute onset gout [16, 32]. In our study, the level of orotic acid and orotidine was increased in the M group relative to the N group, indicating that pyrimidine metabolism was an influencing factor in hyperuricemia; moreover, uric acid, pantothenic acid, and galactonic acid were significantly increased in the M group. Contrary to expectation, the Fa group showed significantly reduced level of orotic acid, orotidine, uric acid, pantothenic acid, and galactonic acid, indicating that pyrimidine metabolism is related to pantothenate and CoA biosynthesis, pentose and glucuronate interconversions, and purinergic metabolic pathways via uracil. These results indicated that CTG can regulate uracil, a nodal molecule in the metabolic networks, to mediate other pathways' regulation with final outcome of decrease in the uric acid level.

Phenylpyruvic acid is a dicarbonyl compound, a product of phenylalanine metabolism. Phenylalanine is an essential amino acid, comprising mostly of tyrosine catalyzed by phenylalanine hydroxylase, and is involved in the synthesis of important neurotransmitters and hormones together with tyrosine as part of the body's glucose and lipid metabolism. Under physiological conditions, only a small proportion of phenylalanine is converted to phenylpyruvic acid under the action of aminotransferases. In case of decreased or lost phenylalanine hydroxylase activity, a large amount of phenylpyruvic acid is generated, which may lead to phenylketonuria, affecting brain development, causing mental retardation and nervous system symptoms such as microcephaly and convulsions. Jiang and Liu reported that both hyperuricemia and gout were associated with the phenylalanine metabolism [7, 16]. In our study, the level of phenylpyruvic acid was significantly increased in the M group, while the disordered phenylalanine metabolism was reversed in the Fa group, indicating that CTG can be used to lower the level of uric acid by regulating the phenylalanine metabolism. Phenylpyruvate can further produce phenyl-acetaldehyde, which subsequently generates phenyl-acetal-CoA, which is associated with pantothenate and CoA biosynthesis via acetyl-CoA.

Chenodeoxycholic acid (CDCA) is closely related to cholesterol. The combination of CDCA with glycine or taurine in the liver can inhibit the cholesterol synthesis, increase the bile secretion in patients with gallstone disease, and be used to treat cholesterol gallstone disease. Previous studies have shown that CDCA has a wide range of effects on the lipid metabolism; hence, it may be associated with the onset of hyperuricemia and gout [33]. In our study, the CDCA content of the model group was lower than that of the normal group, while that of the Fa group was significantly increased. Collectively, these results indicated that hypercholesterolemia can increase the risk of hyperuricemia and gout, while CTG can reduce the level of uric acid through its cholesterol lowering effect.

In our study, pantothenate and CoA biosynthesis was second core of the hyperuricemia metabolic networks. Pantothenic acid, also known as vitamin B5, is one of the 13 essential vitamins in humans. It consists of pantoic acid and *β*-alanine, which is an acyl transporter and participates in the metabolism process, regulating energy and fat metabolism [34]. Acetyl-CoA, an acetylated form of CoA, is a precursor of substances such as fatty acids and ketone bodies and plays a pivotal role in the energy metabolism in vivo [35], whereas CoA can form acyl carrier protein and further produce R-4-'phospho-pantothenate which is associated with pantothenic acid. Acetyl-CoA may be associated with the bile secretion and phenylalanine metabolism. Both vitamin B and acetyl-CoA are involved in the metabolism of sugars, proteins, and lipids in vivo and, thus, may be involved in the development of hyperuricemia; therefore, we hypothesized that the onset of hyperuricemia is associated with vitamin B5 and acetyl-CoA metabolism. In this study, we observed an increase in the level of pantothenic acid in the model group, indicating dysregulated pantothenate and CoA biosynthesis. Moreover, in the M group, the level of chenodeoxycholic acid was decreased and that of phenylpyruvic acid was increased. After CTG intervention, in the Fa group, the level of phenylpyruvic acid was significantly reduced, and that of chenodeoxycholic acid was significantly increased, indicating intersection of pantothenate and CoA biosynthesis with the pathways of pyrimidine metabolism, bile secretion, and phenylalanine metabolism through acetyl-CoA. Thus, CTG affects acetyl-CoA, the nodal molecule in the metabolic networks, to regulate the other pathways with the final outcome of decreased uric acid level.

Among the metabolic diseases such as hypertension, diabetes, obesity, and hyperlipidemia, hyperuricemia is an independent risk factor for several other metabolic diseases. Studies have demonstrated involvement of other inflammatory factors in their pathogenesis. CTG contains Pseudobulbus Cremastrae Seu Pleiones, whose effective ingredient is colchicine, and can significantly reduce inflammation. It is likely that CTG may regulate the metabolism of carbohydrates, amino acids, lipids, vitamins, purines, and pyrimidines to achieve anti-inflammatory effects, reduced uric acid levels, and renal protection.

## 5. Conclusion

In this study, the hyperuricemia rat model was successfully prepared by means of oral gavage of hypoxanthine and intraperitoneal injection of oxonic acid potassium for 10 consecutive days. The levels of SUA, Scr, BUN, and inflammatory cytokines such as TNF-*α* and IL-1*β* were evaluated; the result of serum metabonomics study through UPLC-MS indicated total seven related metabolites including chenodeoxycholic acid, orotic acid, orotidine, uric acid, phenylpyruvic acid, pantothenic acid, and galactonic acid. Hyperuricemia is mainly associated with metabolism of cholesterol, amino acid, lipids, vitamin, and glucose. By regulating the bile secretion, pyrimidine, purine, and phenylalanine metabolism, pantothenate and CoA biosynthesis, and pentose and glucuronate interconversions pathways at various degrees, CTG showed effectiveness to achieve anti-inflammation, lowered level of uric acid in the blood, and protection of the renal function.

## Figures and Tables

**Table 1:** Constituents of Compound Tufuling Granules.

Components	Part used	Amount used (g)
*Rhizoma Smilacis Glabrae*	Root	35
Rhizoma Dioscoreae Hypoglaucae	Root	18
Pseudobulbus Cremastrae Seu Pleiones	Root	15
Semen Vaccariae	Seed	10
Radix Achyranthis Bidentatae	Root	10

**Table 2:** Potential related metabolites and their metabolic pathways.

VIP	Compounds	Formula	Retention time	Measured	Adducts type	M∗	Fa^#^	B^#^	Related pathway
1.26	Galactonic acid	C6H1207	0.65	195.0505	M-H	↑	↓		Pentose and glucuronate interconversions
2.11	Pantothenic acid	C9H17N05	1.71	218.1029	M-H	↑	↓	↓	Pantothenate and CoA biosynthesis
2.13	Orotidine	C10H13N2011P	0.68	287.0516	M-H,2M-H	↑	↓	↓	Pyrimidine metabolism
1.02	Orotic acid	C5H4N204	0.71	155.0094	M-H	↑	↓		Pyrimidine metabolism
12.17	Uric acid	C5H4N403	0.71	167.0207	M-H, 2M-H	↑	↓	↓	Purine metabolism
2.58	Phenylpyruvic acid	C9H803	1.79	163.0397	M-H	↑	↓	↓	Phenylalanine metabolism
4.72	Chenodeoxycholic acid	C02528	4.99	391.285	M-H, 2M-H	↓	↑	↑	Bile secretion

M: model group; N: normal group; Fa: CGT group; B: allopurinol group.

Variable importance in the projection (VIP) was obtained from the OPLS-DA model.

*∗*Compared with the N group, ^∗^p<0.05.

#Compared with the M group, #p<0.05. ↑, Relative increase in signal; ↓, relative decrease in signal.

## Data Availability

The raw data of this article is reliable. Anyone can find the original data through this link https://figshare.com/s/5b323dcee664cbfc8d6d.
